# An Ultrasensitive PCR-Based CRISPR-Cas13a Method for the Detection of *Helicobacter pylori*

**DOI:** 10.3390/jpm12122082

**Published:** 2022-12-17

**Authors:** Yaxuan Wang, Liyang Liu, Xiaochuan Liu, Kai Wu, Xiaoyan Zhu, Liyan Ma, Jianrong Su

**Affiliations:** 1 Department of Clinical Laboratory, Beijing Friendship Hospital, Capital Medical University, Beijing 100050, China; 2 Department of Gastroenterology, Jingdong Medical Area, General Hospital of Chinese PLA, Beijing 101149, China; 3 Department of Gastroenterology, Emergency General Hospital, Beijing 100028, China; 4 Department of Gastroenterology, The Eighth Medical Center of PLA General Hospital, Beijing 100091, China; 5 Department of Clinical Laboratory, The First Affiliated Hospital of Kunming Medical University, Kunming 650032, China

**Keywords:** CRISPR-Cas13a, fluorescence, *Helicobacter pylori*, in vitro transcription, nucleic acid detection, PCR

## Abstract

The rapid and simple detection of *Helicobacter pylori* (*H. pylori*) is essential for its clinical eradication. Although various methods for detecting *H. pylori* have been well established, such as endoscopy in combination with histology or culture, rapid urease test (RUT) and molecular tests using clinical specimens, it is of great importance to develop an ultrasensitive and accurate nucleic acid detection platform and apply it to identify *H. pylori*. To meet these demands, a novel method based on PCR and CRISPR-Cas13a, called PCR-Cas13a, was developed and validated using the DNA of 84 clinical strains and 71 clinical specimens. PCR primers for the pre-amplification of conservative sequence and CRISPR RNA (crRNA) for the detection of specific sequence were designed according to the principle. The designed primers and crRNA were specific to *H. pylori*, and the assay showed a high degree of specificity compared with other common pathogens. Our detection system can screen *H. pylori* with a limit of 2.2 copies/μL within 30 mins after PCR amplification. Using a coincidence analysis with traditional methods, our method exhibited 100% accuracy for the detection of *H. pylori*. Furthermore, its diagnostic performance was compared, in parallel with a q-PCR. The PCR-Cas13a demonstrates 98% sensitivity and 100% specificity. Moreover, our approach had a lower limit of detection (LOD) than q-PCR. Herein, we present a diagnostic system for the highly sensitive screening of *H. pylori* and distinguish it from other pathogens. All the results demonstrated that this PCR-based CRISPR assay has wide application prospects for the detection of *H. pylori* and other slow-growth pathogens.

## 1. Introduction

*Helicobacter pylori* (*H. pylori*) infection was listed in the 15th Report on Carcinogens released by the U.S. Department of Health and Human Services (HHS). It remains a major global public health challenge and may cause serious gastrointestinal diseases, including gastric and duodenal ulcers, mucosa-related tissue lymphoma and gastric cancer [1]. The robust and accurate detection of *H. pylori* is a prerequisite for effective treatment. Numerous methods are available today to detect *H. pylori*, including non-invasive methods, which do not require endoscopy or biopsy specimens (antibody detection from serum and urine, urea breath test [UBT], stool antigen test, and PCR from stool), as well as invasive tests, which require biopsy specimens obtained via endoscopy (histopathology, rapid urease test [RUT], culture, and PCR from biopsy specimens). These methods have many limitations, e.g., they are time consuming and have low specificity or sensitivity. Thus, promising diagnostic tests for *H. pylori* infection should be available with high sensitivity, high specificity, cost-effectiveness and rapid performance, depending on the clinical situation.

Clustered Regularly Interspaced Short Palindromic Repeats (CRISPR) and CRISPR- related proteins (Cas) is a unique RNA-guided adaptive immune system that widely exists in bacteria and archaea [2]. Clustered Regularly Interspaced Short Palindromic Repeats systems recognize foreign nucleic acids based on their sequence and subsequently eliminates them through endonuclease activity associated with the Cas enzyme. Although different CRISPR–Cas systems exist in archaea and bacteria [3], these systems are dependent on CRISPR RNA (crRNA), which directs Cas proteins to recognize and cleave nucleic acid targets. By hybridizing the crRNA to a complementary sequence, the crRNA can be directed toward specific DNA or RNA regions of interest. In some systems, this is restricted to the proximity of a protospacer adjacent motif (PAM) or protospacer flanking sequence [4]. 

The commonly used molecular diagnostic methods for emerging pathogens are mNGS and q-PCR [5,6]. Using mNGS, all nucleic acids in the sample were analyzed to directly identify infectious microorganisms [7]. However, this method has a long detection time and high cost. Currently, q-PCR is recognized as the gold standard of nucleic acid amplification experiments [8,9]. It is important for determining the pathogens of emerging infectious diseases, but it struggles to detect samples containing a low copy of nucleic acids. The CRISPR-based diagnostics could be able to fulfill these unmet needs, and it is superior to other testing methods in terms of time and cost for detecting pathogens associated with emerging infectious diseases [10]. In the field of nucleic acid diagnostics, Cas13a-based detection methods rely on the activation of Cas13 nucleases by guide-RNA to induce non-specific single-stranded RNA to release a caged reporter molecule [11,12,13]. Using a fluorescence detector, the released reporter can be quantitatively measured. Therefore, Cas13 [11,13] is commonly used for pathogen detection [12,14,15,16]. Leptotrichia buccalis Cas13a (LbuCas13a), which exhibits great RNA-guided RNase activity among the Cas13a protein family, is widely used to detect miRNAs, such as miR-17, miR-10b, miR-155 and miR-21 [17,18,19,20,21]. In addition, LbuCas13a is also applied to screen pathogens including SARS-Cov-2, African swine fever virus [22] and *Salmonella enteritidis* [23]. However, its potential use in screening *H. pylori* remains unexplored.

In this study, we established a reaction system for *H. pylori* detection by combining PCR with Cas13a cleavage, which provided a rapid and ultrasensitive molecular method for the diagnosis of *H. pylori* infection. Furthermore, the herein proposed method was successfully applied for detecting *H. pylori* in clinical strains and samples with excellent sensitivity and specificity.

## 2. Materials and Methods

### 2.1. Sample Collection and Genomic DNA Extraction

A total of 66 *H. pylori* clinical strains and 18 other common pathogens, were used in this study. The 66 *H. pylori* clinical strains were isolated from gastric biopsies specimens of patients under endoscopic examination, while the 18 other clinical strains were from the Department of Clinical Laboratory, Beijing Friendship Hospital, including *Escherichia coli* strains, *Staphylococcus aureus* strains, *Enterococcus faecalis* strains, *Enterobacter cloacae* strains, *Staphylococcus saprophyticus* strains and *Klebsiella pneumonia* strains, which were identified by MALDI-TOF MS. The utilized strains were stored in Brucella broth (Solarbio, Beijing, China), including 20% (*w/v*) glycerol (Solarbio, Beijing, China) and 10% bovine serum (Sigma Aldrich, Steinheim, Germany), at −80 °C until bacterial recovery and DNA extraction. All strains except for *H. pylori* were grown on a blood agar plate (Thermo Fisher Scientific, Waltham, MA, USA), and kept in a CO_2_ incubator at 37 °C for 24 h. *Helicobacter pylori* were inoculated onto Columbia blood agar (OXOID, Basingstoke, UK) plates containing 5% fresh defibrinated sheep blood (Solarbio, Beijing, China) with Helicobacter Pylori Selective Supplement (Dent) (OXOID, Basingstoke, UK) under microaerophilic conditions, utilizing a GENbox microaerophilic gas pack (BioMeriuex, Marcy l’Etoile, France) at 37 °C up to 7 days. Genomic DNA was extracted using the TIANamp Bacteria DNA Kit (TIANGEN, Beijing, China), according to the manufacturer’s instruction. 

Seventy-four patients with gastrointestinal diseases who were undergoing an upper gastrointestinal tract endoscopy in Beijing from 2021 to 2022 were included in this study. A biopsy obtained from the antrum or corpus of the stomach of each patient was stored at −80 °C until analysis. It is universally accepted that no single test is considered as the gold standard for *H. pylori* infection diagnosis. In our study, *H. pylori* infection was diagnosed if the RUT and culture tests were both positive [24]. Half of the biopsy was used for RUT and culture, and the other was homogenized in 500 μL of medium (Brucella broth with 20% glycerol) and used for DNA extraction. Then, the genomic DNA was extracted with the QIAamp DNA Mini Kit (Qiagen, Hilden, Germany), following the manufacturer’s instructions. The extracted DNA was quantified by Nanodrop 2000 (Thermo Fisher Scientific, Waltham, MA, USA) and stored at −20 °C for further analysis.

All subjects gave their informed consent for inclusion before they participated in the study. The study was conducted in accordance with the Declaration of Helsinki, and the protocol was approved by the Ethics Committee of Beijing Friendship Hospital, Capital Medical University. (Ethical Approval Number: 2021-P2-199-01).

### 2.2. Oligos and crRNA Preparation

The PCR primers, crRNAs and q-PCR probe used in this study are listed in Table 1, and synthesized by Tianyi Huiyuan Co., Ltd., (Beijing, China). For designing the PCR primers and crRNAs, *H. pylori* sequences downloaded from Nucleotide database were selected as reference sequences (File S1). The nucleotide sequences of *H. pylori* were aligned to identify the conserved regions. A bioinformatics analysis of the *H. pylori* sequences using Clustal X software and Perl scripts were performed to determine the ratio of bases at each location. The crRNAs were designed for subsequent *H. pylori* Cas13a assays in conserved sequences. Three crRNAs were performed by our method for screening the optimal crRNA. Using the Primer Premier 6 software (Premier Biosoft, Palo Alto, CA, USA) and Basic Local Alignment Search Tool (BLAST) database, the PCR primers were designed based on the optimal crRNA. Two crRNA primers were annealed to double-stranded DNA by using Annealing Buffer for DNA Oligos (Solarbio, Beijing, China). The double-stranded DNA was purified by the Universal DNA Purification Kit (TIANGEN, Beijing, China). According to the instructions of the HiScribe T7 Quick High Yield RNA Synthesis Kit (NEB, Ipswich, MA, USA), the double-stranded DNA was incubated at 37 °C overnight and transcribed to crRNA. The crRNA was then purified using RNA Clean & Concentrator-5 (Zymo, Irvine, CA, USA), according to the manufacturer’s instructions, and stored at −80 °C.

### 2.3. Verification of LbuCas13a Activity

Detection was performed as follows: 0.5 μL of Rnase Inhibitor (Takara, Otsu, Japan), 100 nM of LbuCas13a (Bio-lifesci, Guangzhou, China), 100 nM of crRNA, 500nM of quenched fluorescent RNA reporter (Bio-lifesci, Guangzhou, China) and 25 ng of target single-stranded RNA (ssRNA) in 1× reaction buffer (10 mM of Tris-HCL, 50 mM of KCL, 1.5 mM of MgCL_2_). The reactions were conducted at 37 °C for 30mins and monitored on the ABI 7500 Fast Real-Time PCR System (Applied Biosystems, Foster City, CA, USA) every 1 min. The target ssRNA, crRNA or LbuCas13a was replaced by DEPC-treated water (Solarbio, Beijing, China) in the detection system and served as a negative control.

### 2.4. PCR-Cas13a Assay

The T7 promoter sequence (TAATACGACTCACTATAGGG) was appended to the 5′-end of the PCR forward primer. PCR was carried out in a total 50 μL of reaction mixture containing 25 μL Premix Taq (Ex Taq^™^ Version 2.0 plus dye), 0.4 μM of each primer, 2 μL of target DNA and 19 μL of DEPC-treated water with Premix Taq (Ex Taq Version 2.0 plus dye) (Takara, Otsu, Japan). The reaction was performed via an ABI Verti 96-well thermal cycler (Applied Biosystems, Foster City, CA, USA), and the thermal cycle program was as follows: 5 min denaturation at 95 °C proceeded by 35 cycles of 95 °C for 30 s, 60 °C for 30 s, and 72 °C for 15 s, 1 cycle of 72 °C for 10 min, and ending at 4 °C.

For the PCR-Cas13a assay, the reaction system consisted of 20 μL containing 80 nM of crRNA, 80 nM of LbuCas13a, 400nM of quenched fluorescent RNA reporter, 1× reaction buffer, 1.2 μL of NTP Buffer Mix (NEB, Ipswich, MA, USA), 0.4 μL of T7 RNA Polymerase (NEB, Ipswich, MA, USA), 0.4 μL of Rnase Inhibitor, 3 μL of PCR products and 8.2 μL of DEPC-treated water. The reactions were performed at 37 °C for 30 min and monitored on the ABI 7500 Fast Real-Time PCR System. The fluorescence intensity was recorded every 1 min. As a negative control, the reaction system was added with DEPC-treated water instead of the PCR products.

### 2.5. Sensitivity and Specificity of the PCR-Cas13a Fluorescence Detection

To determine the sensitivity of the PCR-Cas13a assay, the genomic DNA of *H. pylori* (ATCC 43504) was extracted using a TIANamp Bacteria DNA Kit; the initial DNA concentration was 40 ng/μL and serially diluted. Different dilutions of the DNA as a template were tested by PCR-Cas13a assay. As a negative control, the reaction system was added with DEPC-treated water instead of the PCR products.

*Escherichia coli* (ATCC 8739), *Staphylococcus aureus* (ATCC 29213), *Enterococcus faecalis* (ATCC 29212), *Enterobacter cloacae* (ATCC 700323), *Staphylococcus saprophyticus* (ATCC BAA-750), *Klebsiella pneumonia* (ATCC 700603) and *H. pylori* (ATCC 43504) were used for specificity determination of the PCR-Cas13a assay. Genomic DNA was extracted using a TIANamp Bacteria DNA Kit. The extracts were tested by the PCR-Cas13a assay.

### 2.6. Q-PCR Assay

Genomic DNA extracted from gastric mucosa was detected to determine the sensitivity and specificity of the q-PCR assay, and the serially diluted genomic DNA of *H. pylori* (ATCC 43504) was tested to determine the assay’s limit of detection (LOD). The details of the q-PCR reaction system are as follows: 10 μL of Premix Ex Taq (Probe qPCR) (2X) (Takara, Otsu, Japan), 0.4 μL of each primer (10 μM), 0.8 μL of the probe reporter (2 μM), 0.2 μL of ROX Reference Dye II (50X) (Takara, Otsu, Japan), 2 μL of DNA templates and 6.2 μL of DEPC-treated water up to 20 μL. Assays were conducted on the ABI 7500 Fast Real-Time PCR System, followed by denaturation at 95 °C for 20s, and 40 cycles consisting of 95 °C for 3 s, followed by 60 °C for 30 s. A negative control was included, in which DEPC-treated water was used in place of the DNA template.

### 2.7. Statistical Analysis

To eliminate experimental error, all assays were carried out in triplicate. The data obtained from the ABI 7500 Fast Real-Time PCR System were transferred to the GraphPad Prism8 (GraphPad, Inc., San Diego, CA, USA) for analysis. If the sample’s fluorescence was statistically significantly different from that of the negative control or higher than 3 times its standard deviation, it was determined to be positive. If, however, the sample’s fluorescence signal did not significantly increase and had no statistically significant difference from the negative control, it was considered to be negative. The Receiver Operator Characteristic (ROC) curve and Cohen′s Kappa were performed by the SPSS 26.0 (SPSS, Chicago, IL, USA). Matthew’s Correlation Coefficient (MCC) and balanced-accuracy were calculated based on the True Positive (*TP*), True Negative (*TN*), False Positive (*FP*) and False Negative (*FN*). The formulas are as follows:

$$\mathrm{MCC}=\frac{TP\times TN-FP\times FN}{\sqrt{\left(TP+FP\right)\times \left(TP+FN\right)\times \left(TN+FP\right)\times \left(TN+FN\right)}};\;\mathrm{balanced}-\mathrm{accuracy}=\frac{1}{2}\times \left(\frac{TP}{TP+FN}+\frac{TN}{TN+FP}\right).$$

## 3. Results

### 3.1. Optimization of the CRISPR-Cas13a Reaction

**Figure 1:**
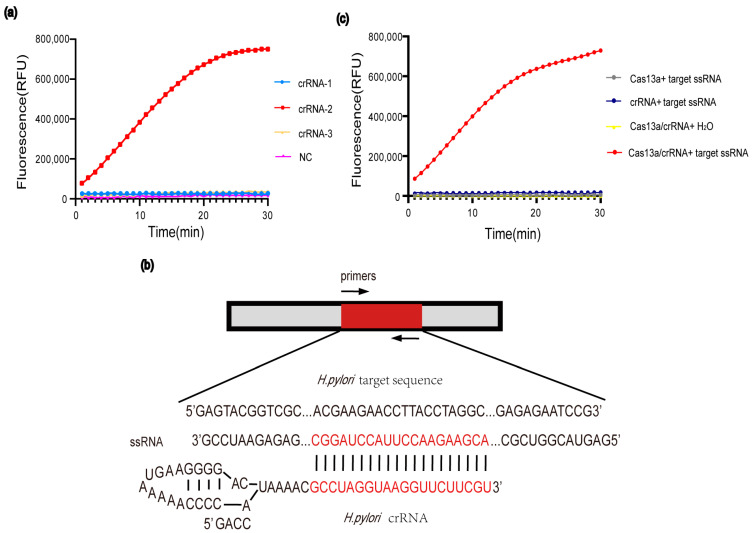
Development of the CRISPR-Cas13a assay. (**a**) To screen efficient crRNA, we performed CRISPR-Cas13a assay by using three designed crRNAs. (**b**) Design of crRNA based on the target sequence of *H. pylori*. (**c**) Activity detection of LbuCas13a.

The optimized crRNA for the CRISPR-Cas13a system was evaluated by fluorescence signal detection. To perform fluorescence detection, 25 ng of ssRNA was introduced to the detection system, and the reaction system was incubated at 37 °C for 30 min. The results indicated that crRNA-2 generated a significant fluorescence signal. Thus, crRNA-2 was chosen and applied for the PCR-Cas13a system (Figure 1a), and the high-quality crRNA that was complementary to the target ssRNA was synthesized by transcription and purification (Figure 1b). 

Further, we evaluated the effect of critical variables such as the LbuCas13a protein, the crRNA and the target ssRNA on the CRISPR-Cas13a detection system. Without target ssRNA, crRNA or LbuCas13a in the reaction system, the background fluorescence was significantly lower than that of the intact reaction system (Figure 1c). The results showed that only in the presence of target ssRNA and Cas13a/crRNA complex could the RNA reporter be efficiently cleaved and release fluorescence signals. 

### 3.2. Establishment of the PCR-Cas13a Detection System 

**Figure 2:**
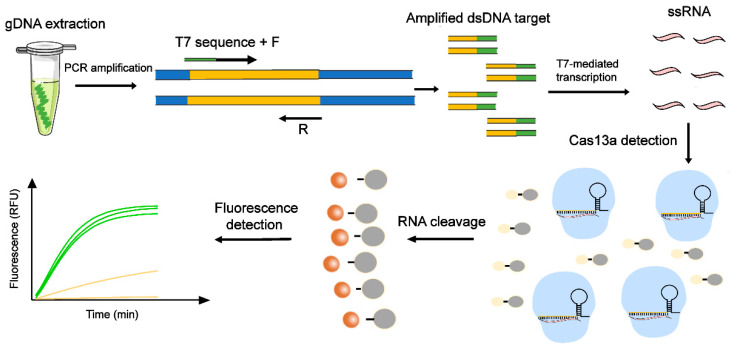
Schematic diagram of the workflow of the PCR-Cas13a system. The extracted genomic DNA was added into the PCR reaction for amplification. The binding of the Cas13a/crRNA complex to the ssRNA transcribed by amplified *H. pylori* DNA targets triggered the collateral activity of Cas13a, which cleaved RNA reporters. The cleaved RNA reporters were detected by fluorescence signals.

In the PCR-Cas13a detection system, the PCR products were transcribed to the target ssRNA using T7-mediated in vitro transcription. The target ssRNA, which binds to crRNA, worked as an activator to trigger the collateral cleavage activity of the Cas13a/crRNA complex. A universal single-stranded fluorescent RNA reporter was employed as the indicator to prove the presence of the target ssRNA. The PCR-Cas13a reaction was carried out at 37 °C for 30 min and monitored every 1 min. When the cleavage effect of Cas13a enzyme was activated, these fluorescent probes were cleaved and released fluorescence. Figure 2 depicts the procedure used for the PCR-Cas13a system.

### 3.3. Evaluation of Sensitivity and Specificity of PCR-Cas13a on H. pylori

**Figure 3:**
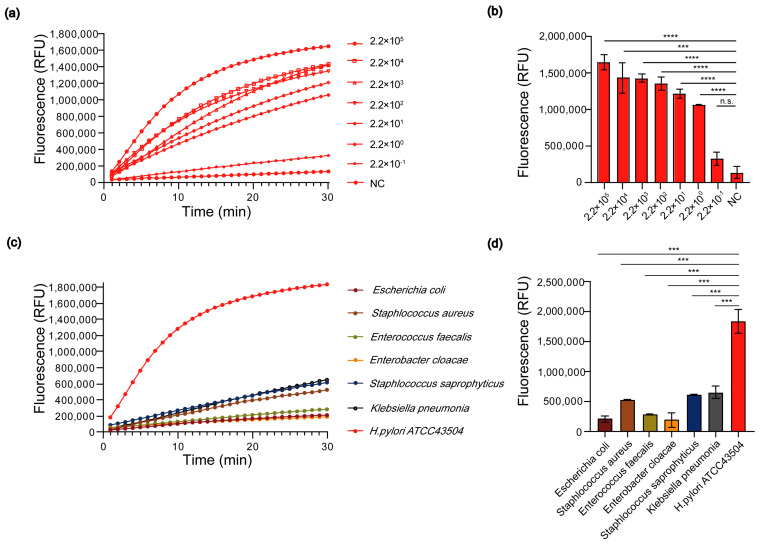
Determination of LOD and specificity of PCR-Cas13a assay. (**a**) Fluorescence curves generated by the PCR-Cas13a reaction at different concentrations of *H. pylori* ATCC 43,504 (2.2 × 10^5^ to 2.2 × 10^−1^ copies/μL). (**b**) Fluorescence values generated by the PCR-Cas13a reaction at different concentrations of *H. pylori* ATCC 43,504 (2.2 × 10^5^ to 2.2 × 10^−1^ copies/μL). ***: *p* < 0.001; ****: *p* < 0.0001; n.s., *p* > 0.05. Data were expressed as mean ± standard deviation (SD). (**c**) Fluorescence curves generated by the PCR-Cas13a reaction at different strains, including *H. pylori* ATCC 43,504 and other common pathogens. (**d**) Fluorescence values generated by the PCR-Cas13a reaction at different strains, including *H. pylori* ATCC 43,504 and other common pathogens. ***: *p* < 0.001. Data were expressed as mean ± standard deviation (SD).

To evaluate the sensitivity of PCR-Cas13a, a series of ten-fold gradient dilutions of *H. pylori* (ATCC 43504) genomic DNA were used as templates for PCR-Cas13a assay. The initial concentration of template DNA amount was 40 ng/μL, which was equivalent to 2.2 × 10^7^ copies/μl (DNA copies number was determined following formula [(6.02 × 10^23^) × genomic DNA concentration (ng/μL) × 10^-9^]/ (genomic DNA length (nt) × 660) = copies/μL). The results showed that fluorescence was observed at concentrations ranging from 2.2 × 10^5^ to 2.2 × 10^0^ copies/μL, indicating that the ultimate LOD of PCR-Cas13a was 2.2 copies/μL (Figure 3a,b). 

DNA extracted from various pathogens was extracted and screened using our detection system. The results showed that the mean fluorescence detection values for *Escherichia coli* (ATCC 8739), *Staphylococcus aureus* (ATCC 29213), *Enterococcus faecalis* (ATCC 29212), *Enterobacter cloacae* (ATCC 700323), *Staphylococcus saprophyticus* (ATCC BAA-750), *Klebsiella pneumonia* (ATCC 700603) and *H. pylori* (ATCC43504) were, respectively, 209,988, 525,417, 283,407, 188,623, 615,364, 648,924 and 1,834,839 (Figure 3c,d). The fluorescence detection values for *H. pylori* (ATCC 43504) could well be discriminated from related common pathogens (*p* < 0.001). The results showed that our method has a high specificity for the detection of *H. pylori*.

### 3.4. PCR-Cas13a Diagnostic Performance

**Figure 4:**
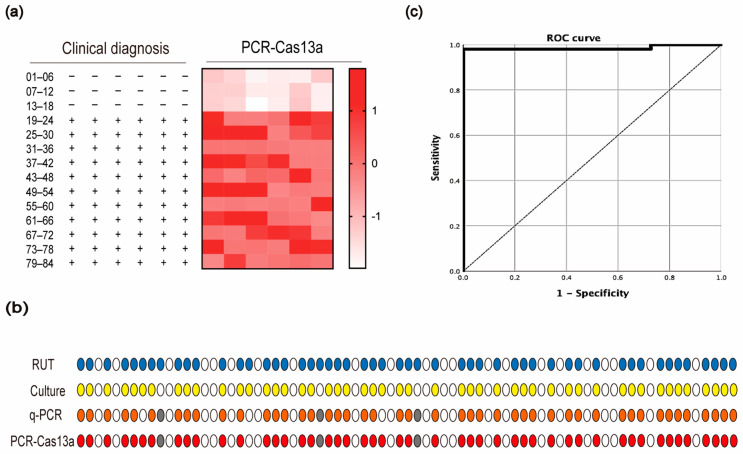
Diagnostic performance of PCR-Cas13a fluorescence assay. (**a**) Detection of *H. pylori* in 84 clinical strains by traditional diagnostic methods (left) and PCR-Cas13a (right). The PCR-Cas13a heatmap represents normalized fluorescence values. (**b**) Comparison of direct detection with gastric specimens from 74 patients. Gray circles represent three samples not used for q-PCR and PCR-Cas13a assay. Circles with other colors (blue for RUT, yellow for culture, orange for q-PCR test, and red for PCR-Cas13a assay) represent positive results, while white circles represent negative results. (**c**) The diagnostic potential of PCR-Cas13a was evaluated by ROC curve analysis with AUC within 95% CI.

To evaluate the efficiency of the PCR-Cas13a system, 66 *H. pylori* isolates and 18 other common pathogens in our laboratory were utilized to compare with traditional diagnostic methods (RUT and culture for *H. pylori*, MALDI-TOF MS for the 18 other strains). As shown in Figure 4a, no statistically significant fluorescence was observed in the other 18 related strains, and all *H. pylori* strains were successfully detected. In addition, the detection results showed that our PCR-Cas13a method had good correlation (84/84, 100% accordance) with traditional diagnostic methods. 

Next, in order to determine the diagnostic performance of the PCR-Cas13a assay, the gastric biopsies from the 71 individuals with gastrointestinal diseases were screened by q-PCR and PCR-Cas13a, in parallel, to compare their sensitivity and specificity. Based on the results of RUT and culture of 74 patients, 49 samples were diagnosed positive for *H. pylori*, 22 samples were detected as negative, and three samples were excluded from our validation assay because the RUT was positive and the culture negative. The specificity was 100% for both methods. In terms of the 49 positive samples, one sample was judged as negative by PCR-Cas13a, while three samples were judged as negative by q-PCR assay (Figure 4b). The sensitivity of the PCR-Cas13a assay for the detection of *H. pylori* in gastric biopsy was 98.0%, which was superior to that of the established q-PCR assay (93.9%) (Table 2). The Area Under Curve (AUC) reached 0.985 (0.956–1 within a 95% confidence interval (CI), *p* < 0.05) based on the ROC curve analysis (Figure 4c). We subsequently calculated other key performance characteristics of the PCR-Cas13a detection assay in clinical samples. The MCC was 0.968, and the balanced-accuracy was 0.990. Moreover, the LOD of PCR-Cas13a was also lower than q-PCR (Figure S1). 

## 4. Discussion

*Helicobacter Pylori* is a Gram-negative microaerophilic bacterium that infects the stomach epithelial cells [26]. A 2017 study revealed that most of the world continues to bear a significant burden of *H. pylori*. Even in Switzerland, which had the lowest recorded *H. pylori* prevalence (18.9%), roughly 1.6 million people were infected. In most developing countries, the prevalence of *H. pylori* is high, and its severity generally correlates with socioeconomic status and hygiene conditions. In China, the *H. pylori* prevalence was 55.8% [27]. *Helicobacter Pylori* has been identified as a carcinogen, and approximately 89% of all gastric cancers may be due to *H. pylori* infection. There is also evidence that the screening and eradication of *H. pylori* in young Chinese adults of China would be cost-effective [28].

In our study, the PCR-Cas13a assay was successfully developed for sensing bacteria, and it demonstrated the specific and sensitive detection of *H. pylori*. This assay can overcome many limitations of the existing methods for *H. pylori* detection and offer a promising molecular alternative for monitoring it.

Currently, each of the conventional methods applied in clinics for detecting *H. pylori* has its own merits and limitations. When conducted under ideal conditions, *H. pylori* culture from gastric biopsy tissues has a sensitivity of over 90% and remains the most specific approach for the bacteria’s detection [29]. Because of the slow growth and microaerophilic conditions of *H. pylori*, this method represents a particular challenge. As for histology, the examination results of this method depend on expert pathologists, and it also essentially related to the number and location of specimens collected [30]. *Helicobacter Pylori* can produce urease, and the RUT is based on detecting urease [31]. *Proteus, Klebsiella, Pseudomonas* and *Staphylococcus* species are the most major urease producers, their presence may influence the results of RUT [32]. Further, the diagnostic performance of the test is determined by the number of bacteria present in the biopsy samples; the UBT is still the most popular non-invasive test for the diagnosis of *H. pylori* infection in clinics. The treatments (PPIs and antibiotics) of *H. pylori* and urease-suppressive may result in false-negative results. Moreover, in order to achieve the highest level of diagnostic accuracy, UBT must be validated and adjusted to account for a variety of factors, including the cut-off value, pretest meal and urea dose. Several antigen preparations, ranging from whole-cell extracts to highly pure single antigens, have been utilized to establish serological tests for the detection of antibodies against *H. pylori*; whole cells or sonicated whole-cell extracts were utilized in early and some later research [33]. The prolonged persistence of antibodies, even after effective eradication therapy, is a specific danger [34], and patients who have been successfully treated may be wrongly asked to repeat therapy on the basis of a positive titer seen only a few weeks after treatment. Although non-invasive tests have the advantages of cost-effectiveness, ease of sampling, and rapid results, their reliability is relatively low due to the low specificity of the antibody detection methods compared to invasive tests. A limitation of conventional techniques, such as the low concentration of *H. pylori* in biopsy samples, is one of the main reasons why this bacterium cannot be detected by culture or other conventional methods. Molecular tests may be superior to traditional methods for identifying *H. pylori*, which are sensitive, rapid and precise techniques for recognizing *H. pylori* in clinical specimens [35,36,37].

There are currently a number of commercially available molecular methods for *H. pylori* and clarithromycin resistance detection, including the *H. pylori* ClariRes, the Allplex *H. pylori* and ClariR, the LightMix^®^*H. pylori* and the *H. pylori* TaqMan^®^ real-time [38]. These methods, which primarily integrate real-time PCR with a melting curve analysis, are highly specific and quick (2 h) molecular approaches that can be used on gastric biopsies and stool specimens [39,40]. Several investigations, however, revealed a very low sensitivity (ranging from 63% to 84%) of *H. pylori* detection from stool specimens using the ClariRes assay, as compared to the stool antigen test and *H. pylori* culture from gastric biopsy specimens [41,42,43]. CRISPR-based diagnostics have primarily focused on detecting pathogens; (mainly, viruses, bacteria and parasites). Viruses that have been detected using CRISPR-based methods include parvovirus B19 [44], Ebola [15], Epstein–Barr virus [45], BK polyomavirus [46] and Coronaviridae (which have become of particular importance during the COVID-19 pandemic). Additionally, CRISPR-based diagnostics have been used to detect bacteria such as *Mycobacterium tuberculosis* [12,47], *Staphylococcus aureus* [12,14], *Listeria monocytogenes* [48], *Pseudomonas aeruginosa* [12] and *Salmonella enteritidis* [23].

A method based on RPA and CRISPR-Cas12a has been developed for the detection of *H. pylori* in stool samples [49]. Compared to Cas12a, Cas13a has an additional-but not tedious-transcriptional procedure. As a result of the transcription process, the PCR amplicon was amplified again, producing a more sensitive detection and a lower LOD. Moreover, Cas13a was found to be more active than Cas12a in terms of trans-cleavage/collateral cleavage, which had a larger linear range and a better signal-to-background ratio for detection [50]. A previous study indicated that the LbuCas13a detection was significantly faster than LbCas12a at low activator concentrations [51]. Additionally, combined with CRISPR type III effector nuclease Csm6, the trans-cleavage signals of Cas13a were enlarged by more than eight-fold [14]. Moreover, compared to RPA, PCR has advantages in the product length, cost and availability of reagent. So far, the majority of CRISPR-based diagnostics has been based on the preamplification of the target. The most commonly used isothermal amplification is RPA. It has been observed that high cost, production delays, the unavailability of TwistAmp nfo kits and the slow response from the technical team may limit the use of RPA technology, since TwistDx relocated to the United States. In addition, the mismatch tolerance property of RPA also generates background amplification noise, which is likely to compete with the target or genuine amplification. Thus, targets should be short, ideally 100–200 bp in length [52]. In general, a large amount of cheap and commercial PCR premix solutions could be used. Further, the preparation of the PCR reaction system is also more convenient and economically advantageous. Furthermore, PCR is less noisy, and amplification lengths are longer and more flexible. In summary, both of these two methods can achieve single-copy LOD. However, PCR technology is more mature and less costly. Additionally, the LOD of our method is expected to be lower when combined with other nucleases.

In this study, based on the alignment results, PCR primer and the crRNAs were designed in the conserved nucleotide region for the detection of *H. pylori*. A robust and ultrasensitive detection method combining PCR reaction and CRISPR-Cas13a was performed as follows: first, the target sequence was amplified by PCR reaction; second, the trans-cleavage activity of CRISPR-Cas13a was activated in the presence of PCR product and T7-mediated transcription system; finally, the screening results were judged on the basis of observing their fluorescence intensity. 

The LOD of another TaqMan q-PCR method established in 2002 was 10^3^ copies/μL [53]. In the study of Javier et al., the LOD of the LightMix^®^ RT-PCR was found at 10^3^ CFUs/ml^39^. When compared to previously reported *H. pylori* screening methods, the detection system developed in this work shows improvement in sensitivity and detection time, as it has a lower LOD of a single copy per microliter for *H. pylori* and the test can be completed within 30 min after PCR amplification. Simultaneously, the test demonstrated no statistically significant fluorescence with several strains of other common pathogens, demonstrating its high specificity, which is critical in nucleic acid testing. Moreover, the sensitivity of our PCR-Cas13a assay across clinical samples exceeded the performance of established real-time PCR, which demonstrates its potential utility in a clinical setting. 

However, there are a few limitations to the PCR-Cas13a assay, despite its excellent results and high sensitivity. First, the method relies upon fluorescence detection, which requires additional instruments, rather than visual readout. By integrating the PCR-Cas13a system with fast or visual detection methods, “fluorescence signals” can be converted into colorimetric signals [54], electrochemical signals [44], lateral flow strips [55], or be combined with microfluidic biosensors [56]. Second, antimicrobial resistance and mutations in drug sensitivity are serious issues in *H. pylori* infection. As a result, we would like to develop a system which can simultaneously identify *H. pylori* and its mutations. 

## 5. Conclusions

Here, we proposed a novel and reliable diagnostic strategy based on PCR combined with CRISPR-Cas13a for the rapid screening of *H. pylori,* which is more ultrasensitive and specific than most of the methods described in this paper. With a suitable target, designed PCR primers and specific crRNA, this method was capable of detecting *H. pylori* specifically and with high sensitivity, and could test 2.2 copies/μl of DNA within 30 min after amplification. 

In conclusion, our CRISPR-Cas13a-based detection system may provide a new and alternative technical approach to identify *H. pylori* infection easier and timely.

## Figures and Tables

**Table 1:** The oligonucleotide sequences used in this study.

Name	Sequence (5′-3′)
PCR-Cas13a	
T7-F1	TAATACGACTCACTATAGGGGAGTACGGTCGCAAGATTA
R1	CGGATTCTCTCAATGTCAAG
crRNA-1	GACCACCCCAAAAAUGAAGGGGACUAAAACUCUCAAUGUCAAGCCUAGGUAAGG
crRNA-2	GACCACCCCAAAAAUGAAGGGGACUAAAACGCCUAGGUAAGGUUCUUCGU
crRNA-3	GACCACCCCAAAAAUGAAGGGGACUAAAACCAAGCCUAGGUAAGGUUCUUCGUG
RNA reporter	FAM-UUUUUU-BHQ1
q-PCR [25]	
F2	CTCATTGCGAAGGCGACCT
R2	TCTAATCCTGTTTGCTCCCCA
Probe	FAM-ATTACTGACGCTGATTGCGCGAAAGC-TAMRA

**Table 2:** Clinical samples consistency comparison between the two detection methods.

		Clinical Diagnosis		Total		Performance Characteristics			
		Positive	Negative		Sensitivity	Specificity	PPV *	NPV *	k
PCR-Cas13a	Positive	48	0	48	98.0%	100%			0.967
	Negative	1	22	23			100%	95.7%	
	Total	49	22	71					
q-PCR	Positive	46	0	46	93.9%	100%			0.905
	Negative	3	22	25			100%	88.0%	
	Total	49	22	71					

* PPV, positive predictive value; * NPV, negative predictive value.

## Data Availability

Not applicable.

## Supplementary Materials

The following supporting information can be downloaded at: https://www.mdpi.com/article/10.3390/jpm12122082/s1, Figure S1: The LOD of the established q-PCR detection method; File S1: The accession number of *Helicobacter pylori* sequences.
