# Supplementary material for: An Ultrasensitive PCR-Based CRISPR-Cas13a Method for the Detection of Helicobacter pylori
Source: J Pers Med. 2022 Dec 17;12(12):2082. doi: 10.3390/jpm12122082 (PMC9784247; doi:10.3390/jpm12122082)
Supplement: Supplementary file 1 [file jpm-12-02082-s001.zip › jpm-2013964-SI.pdf]

Figure S1. The LOD of established q-PCR detection method.

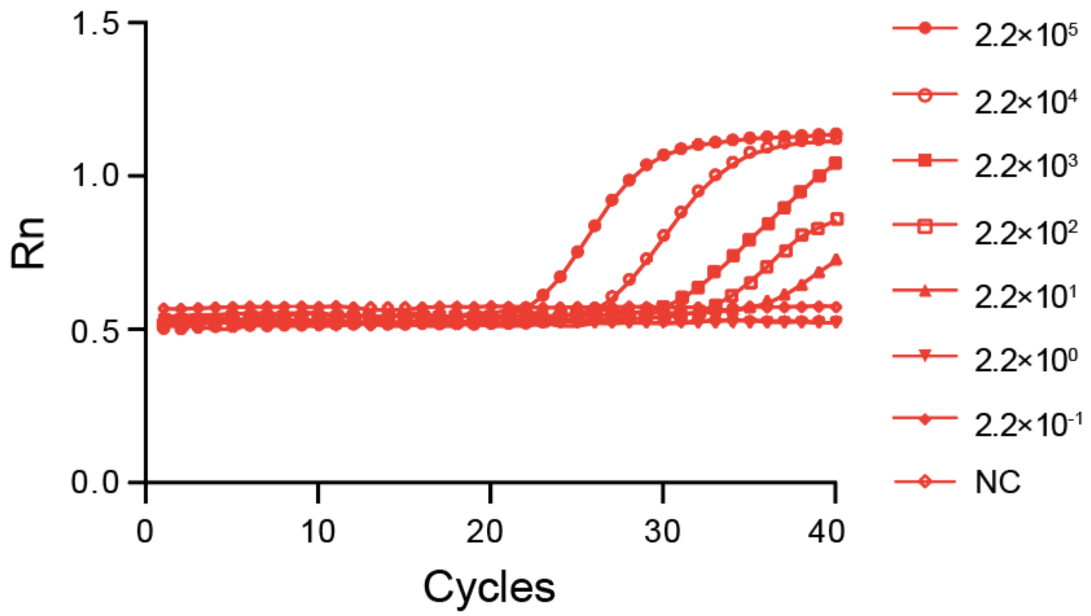

File S1. The accession number of *Helicobacter pylori* sequences：

Z25744.1 Z25742.1 Z25740.1 Z25739.1 Z25738.1 Z25737.1 Z25736.1 Z25743.1 Z25741.1 Z25748.1 CP036398.1 AP017337.1 OP389222.1 OP389221.1 OP389218.1 OP389217.1 OP389216.1 OP225843.1 CP094173.1 CP094172.1 CP094171.1 CP094170.1 CP094168.1 CP094167.1 CP094179.1 CP094165.1 CP094164.1 CP094163.1 CP094161.1 CP036395.1 CP094160.1 CP094159.1 CP094157.1 CP094156.1 CP094153.1 CP094151.1 CP094150.1 CP094149.1 CP094148.1 CP094147.1 CP094145.1 CP094144.1 CP094143.1 CP094142.1 CP094140.1 CP094139.1 CP094138.1 CP094137.1 CP094136.1 CP094135.1 CP094134.1 CP094133.1 CP094132.1 CP094131.1 CP094130.1 CP094129.1 CP094128.1 CP094126.1 CP094124.1 CP094122.1 CP094121.1 CP094178.1 CP094120.1 CP094177.1 CP094175.1 CP094119.1 CP094117.1 CP094116.1 CP094115.1 CP094113.1 CP094111.1 CP094110.1 CP094109.1 CP094108.1 CP094107.1 CP094106.1 CP094105.1 CP094103.1 CP094102.1 CP094101.1 CP094099.1 CP094098.1 CP094096.1 CP094095.1 CP094094.1 CP094093.1 CP094092.1 CP094091.1 CP094090.1 CP094088.1 CP094087.1 CP094085.1 CP094083.1 CP094082.1 CP094080.1 CP094079.1 CP094078.1 CP094076.1 CP094074.1 CP094073.1 CP094071.1 CP094070.1 CP094067.1 CP094066.1 CP094065.1 CP094064.1 CP094063.1 CP094062.1 CP094061.1 CP094059.1 CP094056.1 CP094054.1 CP094052.1 CP094051.1 CP091772.1 CP091771.1 CP091769.1 CP058288.1 CP058286.1 CP058282.1 CP058250.1 CP058286.1 CP058284.1 CP058283.1 CP058281.1 CP058280.1 CP058279.1 CP058252.1 CP058251.1 CP086760.1 OK617321.1 CP071975.1 CP071977.1 CP071978.1 CP071981.1 CP071984.1 CP071985.1 CP071986.1 CP071982.1 CP071987.1 CP051509.1 CP051494.1 CP051533.1 CP051292.1 CP051505.1 CP051509.1 CP051504.1 CP051498.1 CP051540.1 CP051511.1 CP051510.1 CP051508.1 CP051507.1 CP051506.1 CP051503.1 CP051502.1 CP051501.1 CP051499.1 CP051497.1 CP051496.1 CP051541.1 CP051539.1 CP051537.1

CP051493.1 CP051536.1 CP051535.1 CP051435.1 CP051434.1 CP051291.1 CP051290.1 CP053394.1 CP053396.1 CP053392.1 CP053256.1 CP036394.1 CP036393.1 CP036392.1 CP036391.1 CP036390.1 CP036389.1 CP036388.1 CP036387.1 CP036386.1 CP036385.1 CP036384.1 CP036383.1 CP036382.1 CP036381.1 CP036379.1 CP048599.1 CP042211.1 CP032048.1 CP032044.1 CP032043.1 CP032041.1 CP032040.1 CP032039.1 CP032038.1 CP032036.1 CP032034.1 CP032033.1 CP032025.1 CP032024.1 CP032023.1 CP032020.1 CP032046.1 CP032042.1 CP032031.1 CP032037.1 CP032031.1 CP032027.1 CP032022.1 CP024948.1 CP024949.1 CP024951.1 CP024952.1 CP024953.1 CP024946.1 CP024947.1 CP032818.1 CP032912.1 CP032910.1 CP032909.1 CP032906.1 CP032905.1 CP032904.1 CP032903.1 CP032902.1 CP032901.1 CP032900.1 CP032898.1 CP032479.1 CP032478.1 CP032477.1 CP032476.1 CP032475.1 CP032474.1 CP032473.1 CP032472.1 CP032471.1 CP032913.1 CP032908.1 CP032907.1 CP032899.1 CP034147.1 CP036396.1 CP028325.1 CP024072.1 CP024023.1 CP024022.1 CP024021.1 CP024020.1 CP024019.1 CP024018.1 CP024017.1 CP024016.1 CP024079.1 CP024078.1 CP024077.1 CP024076.1 CP024075.1 CP024074.1 CP024073.1 CP024071.1 CP019700.1 CP023267.1 CP023266.1 CP023265.1 CP027021.1 CP007605.1 CP024015.1 CP022409.1 CP006610.2 CP005491.3 CP005490.3 CP006889.1 CP006888.1 CP001582.1 CP011330.1 CP006822.1 CP027020.1 CP002331.1 CP003475.1 CP003476.1 CP048600.1 CP048601.1 CP036397.1 CP036399.1 MN598069.1 MN911416.1 MN588191.1 MN326691.1 MN845953.1 MN386211.1 MN386210.1 MN386209.1 MN386208.1 MN386207.1 MN386206.1 MN386205.1 MN386204.1 MN386203.1 MN386202.1 MN386211.1 MW898313.1 MW599347.1 MW599346.1 MW599345.1 MW320720.1 MT477178.1 MT477177.1 MT160753.1 AP024965.1 AP024962.1 AP024599.1 AP023320.1 AP019730.1 X67854.1 HM046432.1 HM046431.1 HM243135.1 HM099656.1 JN595861.1 M88157.1 AF363064.1 AP023347.1 AP023345.1 AP023344.1 AP017633.1 AP017330.1 AP017355.1 AP017336.1 AP011941.1 AP017333.1 AP024964.1 AP024963.1 LC507450.1 LC507449.1 LC507448.1 LC507447.1 LC589442.1 LC589440.1 NR_114587.1 NR_119304.1 NR_044761.1 LT837687.1 LS483488.1 FN598874.1 AY062899.1 AF535198.1 AF535197.1 AF535196.1 AF535195.1 AF535194.1 AY593991.1 AY593990.1 AY593989.1 AY593988.1 AY593986.1 AY062898.1 AF512997.1 AF302106.1 AY456638.1 AY364437.1 AY304571.1 AY304570.1 KC311711.1 KC311710.1 KC311709.1 KC311708.1 KC311707.1 KC311712.1 AY366421.1 AY394476.1 AY364440.1 AY364439.1 AY155586.1 AF348617.1 KC525433.1 KC525432.1 KC525431.1 MT160752.1 MT160751.1 MT160750.1 DQ202371.1 EU033951.1 EU544199.1 EU544200.1 EU035396.1 HQ266659.1 AY304569.1 AY304551.1 AY155587.1 AY366424.1 AY366423.1 AY366422.1 DQ202383.1 DQ202382.1 DQ202381.1 DQ202380.1 DQ202373.1 DQ202372.1 DQ202379.1 DQ202378.1 DQ202377.1 DQ202376.1 DQ202375.1 DQ202374.1 AKOX01000011.1 UGHQ01000001.1 AKNV01000006.1 AKPI01000001.1 UGHN01000001.1 LFIT01000012.1 LFKG01000012.1 UGJP01000005.1 LFCA01000016.1 LEOV01000028.1 MBIH01000067.1 MILU01000124.1 MILG01000071.1 MIKZ01000096.1 MUOS01000069.1 MUON01000141.1 LFKJ01000001.1 LFKK01000014.1 LFKD01000012.1 LFCB01000022.1 LFBY01000012.1 RPFP01000038.1
